# Deploying Clinical Process Improvement Strategies to Reduce Motion Artifacts and Expiratory Phase Scanning in Chest CT

**DOI:** 10.1038/s41598-019-48423-7

**Published:** 2019-08-14

**Authors:** Ruhani Doda Khera, Ramandeep Singh, Fatemeh Homayounieh, Evan Stone, Travis Redel, Cristy A. Savage, Katherine Stockton, Jo-Anne O. Shepard, Mannudeep K. Kalra, Subba R. Digumarthy

**Affiliations:** 1 Massachusetts General Hospital and Harvard Medical School, MGH Webster Center for Quality and Safety, 55 Fruit Street, Boston, MA 02114 USA; 2 Massachusetts General Hospital and Harvard Medical School, Department of Radiology, 55 Fruit Street, Boston, MA 02114 USA

**Keywords:** Computed tomography, Patient education

## Abstract

We hypothesized that clinical process improvement strategies can reduce frequency of motion artifacts and expiratory phase scanning in chest CT. We reviewed 826 chest CT to establish the baseline frequency. Per clinical process improvement guidelines, we brainstormed corrective measures and priority-pay-off matrix. The first intervention involved education of CT technologists, following which 795 chest CT were reviewed. For the second intervention, instructional videos on optimal breath-hold were shown to 245 adult patients just before their chest CT. Presence of motion artifacts and expiratory phase scanning was assessed. We also reviewed 311 chest CT scans belonging to a control group of patients who did not see the instructional videos. Pareto and percentage run charts were created for baseline and post-intervention data. Baseline incidence of motion artifacts and expiratory phase scanning in chest CT was 35% (292/826). There was no change in the corresponding incidence following the first intervention (36%; 283/795). Respiratory motion and expiratory phase chest CT with the second intervention decreased (8%, 20/245 patients). Instructional videos for patients (and not education and training of CT technologists) reduce the frequency of motion artifacts and expiratory phase scanning in chest CT.

## Introduction

More than any other body region, chest and cardiac computed tomography (CT) are susceptible to motion artifacts from inadequate or suboptimal breath-hold, and the cardiac pulsations^1,2^. Most routine chest CT examinations are performed with a breath-hold in maximal inspiration. This ensures adequate aeration of the lung parenchyma and enables a better evaluation of normal structures and abnormalities. When patients cannot do a good inspiratory breath-hold, suboptimal chest CT examinations result from respiratory motion artifacts and expiratory phase scanning^3^. Suboptimal breath-holding can lead to suboptimal interpretation from the point of view of lesion detection and characterization, over-diagnosis (such as for bronchiectasis and pulmonary embolism), and inability to assess stability or change in lesion size, attenuation or morphology. Repeat CT scanning in patients with suboptimal prior test increases radiation dose, contrast volume burden, and healthcare costs while delaying patient care and impairing patient perceptions of the healthcare delivery systems^4–6^. Aside from these concerns, respiratory motion artifacts and expiratory phase scanning in chest CT with or without repeat CT scanning can have a negative effect on clinical workflow in busy and large practices for both CT technologists and the interpreting radiologists.

The current project stemmed from clinical concerns over the frequency of respiratory motion artifacts and expiratory phase scanning in chest CT in our quaternary healthcare institution which has 18 multi-detector-row CT (MDCT) scanners from three vendors at six different geographic locations, and over 80 full- and part-time CT technologists. The concerns occurred despite an active and instantaneous feedback system in place for communicating suboptimal CT examinations, and written and archived CT protocols and policies for chest CT scanning. To address these concerns, we applied clinical process improvement strategies to reduce the frequency of motion artifacts and expiratory phase scanning in chest CT by 50% compared to the baseline prevalence.

## Results

### Baseline data

Close to a third of the patients (35%; 292/826) in the baseline chest CT data had motion artifacts or were expiratory. Both the inpatients (60%, 152/252) and the outpatients (24%, 140/574) had respiratory motion artifacts or expiratory phase scanning on chest CT.

**Figure 1:**
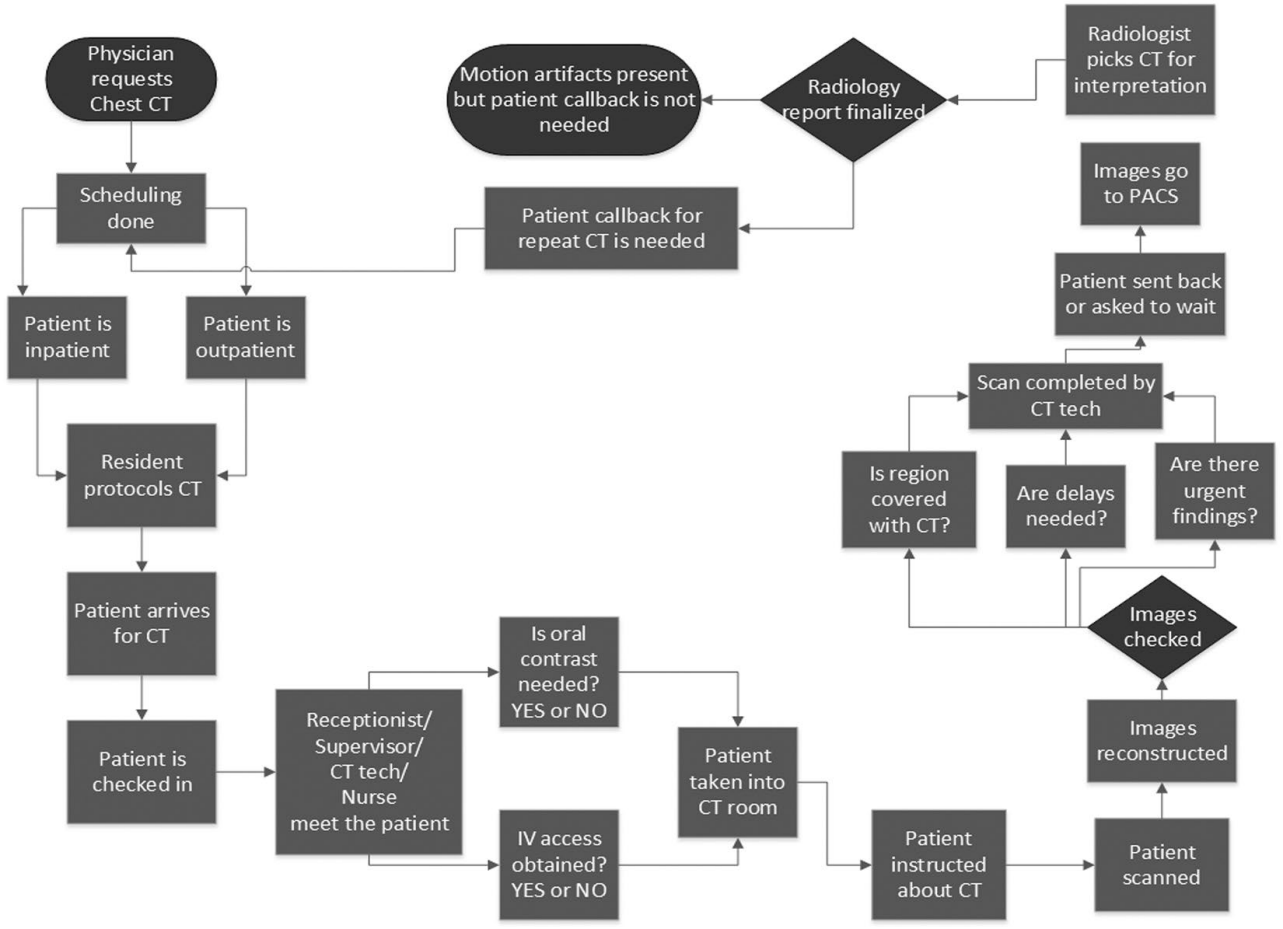
Process Map of Chest CT from physician’s request for a chest CT to finalization of the radiology report.

**Figure 2:**
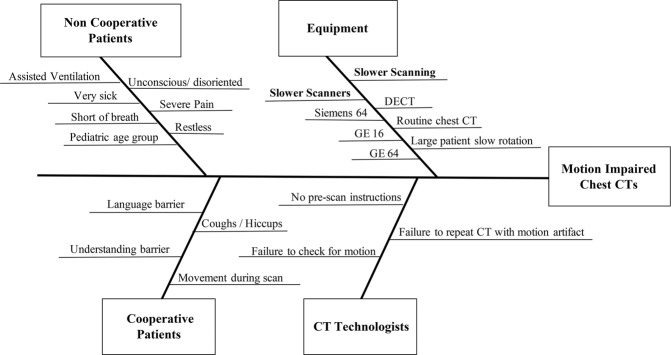
Ishikawa Fishbone Diagram. The cause and effect diagram for motion impaired and expiratory phase chest CT examinations.

**Figure 3:**
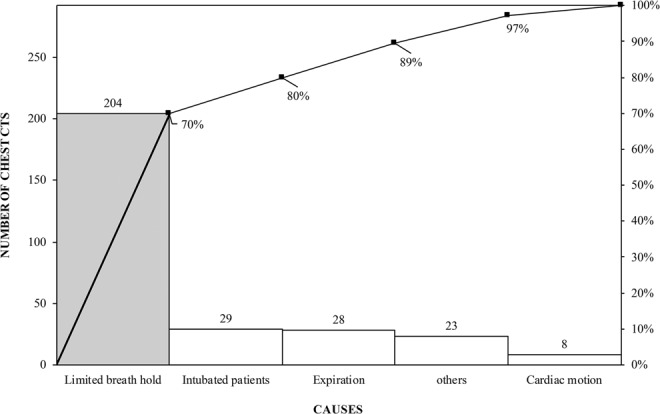
Diagnostic data in Pareto chart depicts the most common causes of motion impaired and expiratory phase scanning in chest CT.

Process map and Ishikawa fishbone diagram are illustrated in Figs 1 and 2. Pareto chart (Fig. 3) summarizes different causes of suboptimal chest CT. The most common cause of suboptimal chest CT was expiratory phase scanning, respiratory motion artifacts, patient intubation leading to expiratory phase scanning or motion artifacts, and cardiac pulsation related artifacts. Table 1 summarizes the priority and payoff matrix for different strategies to reduce respiratory motion artifacts and expiratory phase scanning.

**Table 1:** Priority Pay-off Matrix lists different strategies to reduce respiratory motion artifacts and expiratory phase scanning.

**High impact**	• Handouts with instructions to the patients	• Ask physicians to inform about breath hold ability of patients
	• Videos for patients on breath hold before CT	• Ask radiologists to check and recommend patient call back
	• Ask CT technologist to demonstrate breath hold before CT	• Ask CT tech to check breathing artifacts and repeat CT
	• Teaching module for motion artifacts for CT technologists	• Checklist to triage patients based on breath-hold ability
		• Do all chest CT on faster CT scanners
**Low impact**	• Remove automated patient instructions (API) for chest CT	• Ask CT vendors to create automatic detection of motion artifacts
	• Increase/decrease scan delay following end of API	• Recommend a change of older CT scanners
	• Educate CT technologists about problem	• Avoid scanning patients who will have motion artifacts
	• Give motion grades for performing CT technologists (competition)	• Train radiologists to interpret chest CT with motion artifacts
	**Easy implementation**	**Difficult implementation**

### PDSA-1 and PDSA-2

The frequency of motion artifacts and expiratory phase scanning in the chest CT examinations during PDSA-1 cycle was 36% (283/795). Both the inpatients (53%, 142/267) and outpatients (27%, 141/528) had motion artifacts or expiratory phase scanning. There was no significant statistical change in the frequency of the suboptimal chest CT examinations performed over the PDSA-1 (p = 0.917).

**Figure 4:**
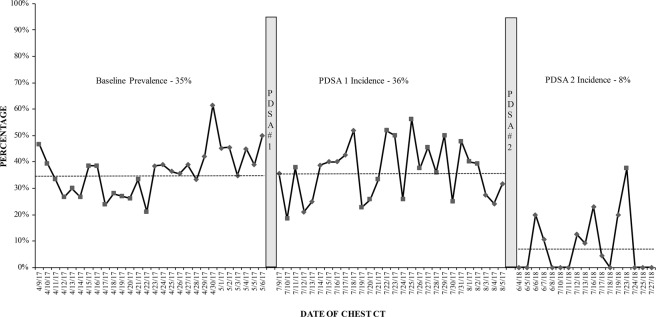
The statistical process chart shows high frequency of motion impaired and expiratory phase chest CT at the baseline and in the PDSA-1 cycle, which reduced substantially in the PDSA-2 cycle.

The magnitude of motion artifacts and expiratory phase scanning in patients who viewed the instructional videos during the PDSA-2 was 8.2% (20/245 patients). Both inpatients (18%, 3/17) and outpatients (7%, 17/228) experienced a reduction in motion artifacts and expiratory phase scanning during the PDSA-2. These reductions in PDSA-2 were significant compared to both baseline data and PDSA-1 (p < 0.0001). Although lower than the baseline and the PDSA-1 data, the frequency of motion artifacts and expiratory phase scanning in the control group patients (15%, 46/311) was significantly greater than those scanned following the instructional videos in the PDSA-2 (p = 0.016). The statistical process chart summarizes the baseline, PDSA-1 and PDSA-2 data (Fig. 4).

All survey patients (100%, 245/245) indicated that they understood the message of the instructional videos. About 88% (215/245) said that the videos helped them follow the breath-hold instructions during their chest CT. Most patients said that they had undergone a CT before (98%, 239/245) which may explain why they were not less anxious after they saw the video (82%, 200/245).

## Discussion

“I hear I forget, I see I remember, I do I understand”. These words from the most famous Chinese teacher, editor, politician and philosopher Confucius (551–479 BC) may explain why the instructional videos that made the patients “see (view)” and “do (practice)” breath hold with the instructional videos in the PDSA-2 cycle helped reduce the frequency of motion- and expiration-impaired chest CT. According to the patient survey, most patients opined that the instructional video helped them follow the breath-hold instructions for chest CT. In the healthcare settings, video modelling can motivate knowledge acquisition, decrease pre-exam anxiety, and enhance self-care^7^.

Though not specific to breath-holding for chest CT, use of instructional videos has been described in radiology. A recent study reported that patient’s ability to relax during cardiovascular MRI increased with addition of video-based information before their exam^8^. However, authors observed no change in the motion artifacts associated with the cardiovascular MRI. This finding is contradictory to our study and may have occurred due to differences in the imaging modalities (CT is much faster than MRI; CT is less often associated with claustrophobia than MRI) and the cause for motion artifacts (respiratory motion in our study versus cardiac pulsations and respiratory motion for cardiovascular MRI). Another study reported that instructional videos for informing patients about the advantages, risks, and alternatives to intravenous contrast media for CT are better than routine discussion for getting informed consent^9^. The impact of video-assisted patient education varies with presentation type; videos with real people are more effective in altering patient behavior than videos with audio and graphical representation of health information^10^.

Instructional videos for patients and healthcare staff are used in other medical specialties. In the elderly patients, instructional videos improved comprehension and satisfaction with the pain clinics^11^. A prior publication has reported that self-instruction videos are better than traditional didactic instructions for cardiopulmonary resuscitation in healthcare providers^12^. In fact, the American Heart Association and the American Red Cross use an array of instructor-led and video-based teaching to facilitate learning. Similar to our study, Marini *et al*. improved patient knowledge, satisfaction, and compliance with an informational video on venous thromboembolism prophylaxis. Patients who watched the video gave 83% correct responses as compared to 62% in those who did not^13^. Another study documented enhanced stroke literacy and efficiency in identifying stroke symptoms, as well as patient satisfaction with video based education of hospitalized stroke patients^14^. According to Tuong *et al*., patient educational videos are also more effective than written materials to increase knowledge and modify health behaviors including cancer screening, heart failure self-care adherence, and HIV testing. They also offer the benefits of providing standardized content across learners, are less resource intensive than written materials or educational sessions, and are effective among viewers of low literacy levels for other medical conditions as well^15^. Recently, Beskind *et al*. have reported that an educational video on cardiopulmonary resuscitation (CPR) for high school students increased their CPR responsiveness and performance^16^. Educational videos have also proven worthwhile in resident learning and training in interventional breast procedures such as stereotactic core biopsy of the breast^17^. The benefit noted from patient educational videos in our study concurs with the abovementioned publications on instructional videos in different clinical settings.

While CT technologists are paramount to quality CT examinations, they may not deliver the desired results due to the demands of multi-tasking. The PDSA-1 cycle that involved retraining of our CT technologists for acquiring chest CT with a lower frequency of respiratory motion and expiratory phase scanning was unsuccessful. This could be related to the immense technologist workload which involves checking patient identification, patient positioning, centering, oral and intravenous contrast administration, establishing and checking intravenous access during contrast administration, selecting CT and contrast injection protocols, assessing adequacy of scan coverage, determining need for delayed images, reconstructing and reformatting CT images, pushing the images to the PACS, and completing the exam in the Radiology Information System. Another reason for the PDSA-1 failure might be use of automatic patient instructions for breath-hold in our institution. Bankier *et al*. have reported that the convenient automatic patient instructions reduce image quality and increase the proportion of repeat CT acquisitions^18^. They recommend personal communication with the patients to humanize the high-tech CT environment and reduce patient anxiety. However, this is not possible in our institution due to a heterogeneous, multiethnic CT technologists’ workforce (n > 80 from various backgrounds and accents). This heterogeneity may have contributed to inadequate training and/or insufficient participation of the CT technologists at our institution.

Chief amongst the implications of our study is the fact that patient involvement and education had a greater effect on the quality of chest CT than the CT technologists as noted from the two-fold lower incidence of motion artifacts and expiratory phase scanning amongst subjects in the PDSA-2 cycle who viewed the instructional videos compared to those scanned without video viewing and with technologist training alone. Such patient involvement can be accomplished with instructional videos which we have made available free of cost (Supplementary Videos 1–3). Patients can view these videos in the waiting room or in the scanner room before their scanning. Although the frequency decreased for all patients recruited in the PDSA-2 cycle compared to the baseline and PDSA-1 data, motion artifacts and expiratory phase scanning in chest CT were greater in the inpatients (18%) than in the outpatients (7%). Many inpatients with impaired chest CT due to motion artifacts or expiratory phase scanning had an intubation or a substantial cognitive impairment to understand breath-hold instructions or videos. To address this issue in the inpatients, we instituted fast, non-breath-hold CT protocol (scan duration <2 seconds) for patients who cannot hold their breath for 5 seconds for their chest CT. Routine application of these fast scan protocols is not possible since they are not suitable for larger patients and are incompatible with the chest CT performed with dual energy scanning mode. Since we used our departmental media services to create and edit the instructional videos and deployed the existing computers to display them to our patients, there were no costs associated with the initiative. Users can either download our videos from the above link for free or create their own from any camera or cellular phone device.

Our study has limitations. The baseline frequency of motion artifacts and expiratory phase scanning in chest CT examinations in our institution may be higher than other imaging sites. This may limit the magnitude of reduction in the frequency noted with instructional videos at sites with different practice type. The failure of PDSA-1 in our institution may have resulted from a heterogeneous group of part-time and full-time CT technologist (n > 80) in a busy CT practice (n > 130,000 CT examinations each year). In smaller and less complicated practices, adequate training of the CT technologists may reduce the frequency of motion- and expiration-impaired chest CT. Although the instructional videos are available only in English, we are working on translating the video into other languages. The frequency of motion- and expiration-impaired chest CT amongst inpatients remain high since many did not or could not follow the instructional videos. For such patients, we introduced a fast, non-breath-hold scanning protocols on several MDCT scanners. However, our CT technologists did not apply these fast protocols in eligible patients who did not follow breath-hold instructions. Although our study does not have a prolonged washout phase to assess the lasting effect of the PDSA-2, the patients participating in the PDSA-2 were recruited over a period of 2 months. Although we did not compare our methods of addressing motion- and expiration-impaired chest CT with other methods of reducing their frequency, our CT technologists have used the other methods of coaching and interacting with the patients. For example, our technologists instructed the patients to follow the breath-hold instructions prior to their scanning. Patients were also provided automatic voice instructions at the time of scanning to help them with the breath-hold.

In conclusion, as compared to education and training of CT technologists, patient instructional videos led to a substantial reduction in the frequency of motion artifacts and expiratory phase scanning in chest CT examinations. Clinical process improvement beyond caregiver education and training are necessary to reduce frequency of motion impaired and suboptimal expiratory phase chest CT.

## Methods and Materials

Per the hospital policy, the Partners Human Research Committee (PHRC) exempted this quality assessment and improvement project from the need for approval. All methods were performed in accordance with the relevant guidelines and regulations. The MDCT scanners included in our study ranged from 64-slice and 256 single-source (GE VCT, Discovery 750 HD and Revolution, GE Healthcare, Waukesha, WI), 128-slice and 192-slice dual-source (Siemens Flash and Force, Siemens Healthineers, Forchheim, Germany), and 128-slice single-source (Philips iCT, Eindhoven, Netherlands). The scanners and the scan protocols for chest CT did not change over the course of the study.

### Baseline data

Baseline data included 826 consecutive chest CT examinations (mean age 62 ± 16 years; 421 women, 405 men) performed between April 9, 2017 and May 6, 2017. These examinations belonged to both inpatients and outpatients. We reviewed the CT images and the radiology reports in the picture archiving and communication system (PACS) for presence of respiratory motion artifacts and expiratory phase imaging that limited evaluation of normal structures or abnormalities in the thorax. Chest CT examinations with motion artifacts confined to the para-cardiac regions, likely related to cardiac pulsations were deemed optimal. For each examination, we recorded the date and time of CT, patient age, gender, endotracheal intubation, location at the time of scanning (inpatient or outpatient), study indications, pertinent radiology report findings, and any mention of respiratory motion artifacts and expiratory phase scanning in the radiology reports.

### Process and cause analysis

In accordance with the process improvement steps, we determined the frequency and probable causes of respiratory motion artifacts and expiratory phase scanning in the baseline chest CT examinations^19,20^. We created a Pareto chart with the QI Macros software (KnowWare International, Inc., Denver, CO). A detailed process map was created from physician order entry for chest CT up to the radiology report finalization (Microsoft Visio, Microsoft Inc., Redmond, WA). To understand the sources and the causes of motion artifacts and expiratory phase scanning in chest CT, we created an Ishikawa fishbone diagram using the Visio program.

Next, we conducted two brainstorming sessions under leadership of two senior chest subspecialty radiologists (SD with 16-year experience, MK with 13-year experience in thoracic imaging). In the first session, these radiologists discussed causes and strategies to mitigate respiratory motion artifacts and expiratory phase scanning with CT quality assurance manager, CT operations manager, senior imaging operations manager, and CT technologist supervisor. Discussions from the first session were brainstormed with the other radiologists in the thoracic imaging division in the second session. A priority and payoff matrix helped stratify various interventions per their ease of implementation and impact.

The outcome measure was defined as the percentage of chest CT with respiratory motion artifacts or expiratory phase scanning. The balance measure was any increase in time required to complete the acquisition of chest CT because of any intervention designed to reduce the frequency of suboptimal chest CT.

### Plan, do, study and act cycle 1 (PDSA-1)

From the priority-payoff matrix, we decided to increase awareness about the frequency, and need for reducing suboptimal chest CT amongst the CT technologists. This was conducted as part of the PDSA-1 in three interactive didactic sessions to educate all CT technologists taking part in the three work shifts. Each session lasted for 30–45 minutes and was led by one of the two senior chest subspecialty radiologists (SD or MK). During these sessions, the radiologists discussed the incidence, cause and implications of suboptimal chest CT from respiratory motion artifacts and expiratory phase scanning. We presented the baseline data on incidence of suboptimal chest CT and demonstrated techniques for enhancing patient cooperation with breath-hold instructions.

After the training sessions of the PDSA-1, we reviewed 795 consecutive chest CT examinations (mean age 62 ± 15 years; 397 women, 398 men) performed between July 9, 2017 and August 5, 2017. A thorough review of both chest CT images and radiology reports was performed to record the above data fields described in the baseline data section.

### PDSA cycle 2 (PDSA-2)

Since the PDSA-1 did not decrease the frequency of motion artifacts and expiratory phase scanning in chest CT, we implemented PDSA-2. We created three instructional videos with role players (co-authors masquerading as a patient and a CT technologist in the actual CT suite) using professional videography services. Informed consent was taken for study participation and publication of identifying information/images in an online open-access publication. These videos showed good breath-hold for chest CT and encouraged patients to practice breath-hold before their scanning. The three video clips demonstrated maximum inspiratory breath-hold for routine chest and diffuse lung disease protocols (Supplementary Video 1), expiratory breath-hold for expiratory phase scanning for diffuse lung disease protocol (Supplementary Video 2), and suspension of breathing without preceding deep breaths for pulmonary embolism protocol (Supplementary Video 3). The participating patients saw protocol-specific video in the scanner suite before their chest CT from June 4th, 2018 to July 27th, 2018. Following their chest CT, patients completed a brief survey on their experience (Table 2).

**Table 2:** Survey questionnaire used in our study for the PDSA-2 cycle.

Survey Questionnaire		
1: Have you undergone a CT of your chest in the past?		
A. Yes	B. No	C. Not sure
2: Did you understand the breathing instructions in the video?		
A. Yes	B. No	C. Not sure
3. Did the video help you follow the breathing instructions in CT scanner?		
A. Yes	B. No	C. Not sure
4. Did the video make you less anxious about having a CT scan?		
A. Yes	B. No	C. Not sure

There were 245 patients (mean age 66 ± 14 years; 126 women, 119 men) in the PDSA-2 who viewed the instructional videos before their chest CT examinations between June 4, 2018 and July 27, 2018. We reviewed chest CT images and radiology reports of all patients and recorded similar data fields described in the preceding baseline data section. Over this timeframe, we also reviewed 311 consecutive chest CT examinations (mean age 64 ± 14 years; 152 women, 159 men) performed in the patients who did not see the instructional videos. These 311 chest CT examinations constituted the control group to assess long-term effect of PDSA-1 and to assess the frequency of suboptimal chest CT during the same time that PDSA-2 was being conducted.

### Statistical analysis

Data were recorded in Microsoft Excel for statistical analyses. A percentage run-chart (p chart) displayed the trend of suboptimal chest CT at the baseline and during the PDSA-1 and PDSA-2 (QI Macros). Descriptive statistics were performed to determine the mean patient age, gender count, and frequency of motion artifacts and expiratory phase scanning in chest CT performed over the duration of the study. We used Wilcoxon rank sum test to compare patients’ frequency of suboptimal chest CT in the baseline data, PDSA-1, PDSA-2, and the control group. A p-value of less than 0.05 was considered as a significant difference.

## Supplementary information

Supplementary Video 1

Supplementary Video 2

Supplementary Video 3

## Data Availability

We will be happy to share de-identified EXCEL data of the study. Actual clinical images cannot be shared due to patient privacy issues.
